# Synthesis by Melt-Polymerization of a Novel Series of Bio-Based and Biodegradable Thiophene-Containing Copolyesters with Promising Gas Barrier and High Thermomechanical Properties

**DOI:** 10.3390/molecules28041825

**Published:** 2023-02-15

**Authors:** Lesly Dasilva Wandji Djouonkep, Christian Tatchum Tamo, Belle Elda Simo, Nasiru Issah, Marc Nivic Tchouagtie, Naomie Beolle Songwe Selabi, Ingo Doench, Arnaud Kamdem Tamo, Binqiang Xie, Anayancy Osorio-Madrazo

**Affiliations:** 1 Department of Petroleum Engineering, Applied Chemistry in Oil and Gas Fields, Yangtze University, Wuhan 430100, China; 2 Lost Circulation Control Laboratory, National Engineering Laboratory for Petroleum Drilling Engineering, Yangtze University, Wuhan 430100, China; 3 Key Laboratory of Drilling and Production Engineering for Oil and Gas, Wuhan 430100, China; 4 National Advanced School of Engineering, University of Maroua, Maroua P.O. Box 46, Cameroon; 5 Department of Earth Sciences, University of Dschang, Dschang P.O. Box 96, Cameroon; 6 Department of Biochemistry, Kwame Nkrumah University, Kabwe P.O. Box 80404, Ghana; 7 Department of Geography, University of Douala, Douala P.O. Box 2701, Cameroon; 8 Institute of Advanced Materials and Nanotechnology, Wuhan University of Science and Technology, Wuhan 430081, China; 9 Laboratory for Bioinspired Materials, Institute of Microsystems Engineering—IMTEK, University of Freiburg, 79110 Freiburg, Germany; 10 Freiburg Center for Interactive Materials and Bioinspired Technologies—FIT, University of Freiburg, 79110 Freiburg, Germany; 11 Freiburg Materials Research Center—FMF, University of Freiburg, 79104 Freiburg, Germany

**Keywords:** thiophene-based polyesters, 2,5-thiophenedicarboxylate, bis(hydroxyalkyl)benzene, aliphatic diols, mechanical properties, biodegradation

## Abstract

Volatile global oil prices, owing to the scarcity of fossil resources, have impacted the cost of producing petrochemicals. Therefore, there is a need to seek novel, renewable chemicals from biomass feedstocks that have comparable properties to petrochemicals. In this study, synthesis, thermal and mechanical properties, and degradability studies of a novel series of sustainable thiophene-based copolyesters like poly(hexylene 2,5-thiophenedicarboxylate-co-bis(2-hydroxyethoxybenzene) (PTB_x_H_y_) were conducted via a controlled melt polymerization method. Fourier-transform infrared (FTIR) and nuclear magnetic resonance (^1^H NMR) spectroscopy techniques elucidated the degree of randomness and structural properties of copolyesters. Meanwhile, gel permeation chromatography (GPC) analysis showed a high average molecular weight in the range of 67.4–78.7 × 10^3^ g/mol. The glass transition temperature (T_g_) was between 69.4 and 105.5 °C, and the melting point between 173.7 and 194.2 °C. The synthesized polymers outperformed poly(ethylene 2,5-thiophenedicarboxylate) (PETF) and behaved similarly to polyethylene terephthalate. The copolyesters exhibited a high tensile strength of 46.4–70.5 MPa and a toughness of more than 600%, superior to their corresponding homopolyesters. The copolyesters, which ranged from 1,4-bis(2-hydroxyethyl)benzene thiophenedicarboxylate (TBB)-enriched to hexylene thiophenedicarboxylate (THH)-enriched, offered significant control over crystallinity, thermal and mechanical properties. Enzymatic hydrolysis of synthetized polymers using porcine pancreatic lipase (PP-L) over a short period resulted in significant weight losses of 9.6, 11.4, 30.2, and 35 wt%, as observed by scanning electron microscopy (SEM), with perforations visible on all surfaces of the films. Thus, thiophene-based polyesters with cyclic aromatic structures similar to terephthalic acid (TPA) show great promise as PET mimics. At the same time, PP-L appears to be a promising biocatalyst for the degradation of bioplastic waste and its recycling via re-synthesis processes.

## 1. Introduction

Petroleum derivatives, including oil and gas, are indispensable in today’s global economy. They play an essential role in the energy transition crisis by providing affordable and reliable energy capacities required by all sectors of transport, and heat engines, including the manufacturing processes of many chemicals and medicines [1,2,3,4]. Overreliance on petroleum resources for the production of polymers and raw materials has largely led to the depletion of accessible fossil reserves and increased carbon emissions through dangerous and economical industrial processes that constantly release toxic materials into the surrounding environment. In addition, the volatility of the prices of oil and its derivatives, combined with the growing demand for plastics, has considerably influenced the prices of the raw materials used in the synthesis of biopolymers and other polymer-based products [5,6,7,8,9,10]. Therefore, research on the design, development and recycling of biomass feedstocks to create substitutes for petro-plastics is imperative to achieving a circular economy and net zero. Researchers are actively turning to these renewable raw materials, such as forest product waste, agricultural residues, organic fractions of municipal solid waste, paper, cardboard, plastic, food waste, green waste, and other waste, because of their affordable prices [11,12,13,14], and as viable alternatives for the preparation of bio-based polyesters with similar to better material properties and environmental friendliness to their petroleum counterparts [15,16,17,18].

Polymeric materials consist of aliphatic polyesters or aliphatic/aromatic polyesters, which may break down more quickly when they reach the end of their useful life. This is because macromolecules have potentially hydrolyzable ester bonds and short aliphatic chains that can be broken down by bacteria, fungi, or algae in the environment [19,20,21].

The different types of biopolymers include sugar-based, cellulose-based, synthetic-based biopolymers, and other natural polymers. In 2022, all of these biopolymers were worth USD 10,979.74 million and were projected to reach USD 31,223.47 million by 2027, growing at a compound annual growth rate (CAGR) of 19.03% during the forecast period [22,23,24,25,26]. Over these years, whether recyclable or not, raw materials from renewable resources have a role to play in environmental protection, disposal, and the safe use of plastics. Some of the more common biopolymers that have been used commercially or are being investigated for commercial applications include poly(lactic acid) (PLA), poly(butylene succinate) (PBS), polyglycolic acid (PGA), polycaprolactone (PCL), polyhydroxyalkanoate (PHA), poly(3-hydroxy valerate), and poly(succinate-co-butylene terephthalate) (PBST) [7,27,28,29]. Unfortunately, while these polyesters derived from aliphatic monomers possess the advantages of short-lived degradation and barrier properties, their strength and storage modulus remain too low to be commercially viable on a large scale, compared to petrochemicals. Thus, a viable strategy to improve the properties of polymers is to blend them with aromatic compounds, to benefit from the synergistic properties of aliphatic and aromatic polyesters offered by various techniques such as chain customization, co-crystallization, melt blending or co-extrusion, nanotechnology, and surface coating to expand their use [30,31,32,33,34]. The resulting biopolymers are incredibly versatile, due to their blend structures, which overcome the shortcomings raised by conventional single-use plastics, such as low flexibility, thermal stability, gas barrier properties, and biodegradability. The process of melt transpolyesterification allows the synthesis of an unlimited mixture of polymer properties, with good material properties obtained through their respective monomers, and with potential application in food packaging or bottle-grade products [35,36].

Polyethylene terephthalate (PET) is one of the most widely used polyesters, due to its unique properties such as durability, cost effectiveness, light weight, and transparency. However, its non-degradable profile and limited barrier to gas properties have drawn criticism of this material in packaging and bottle-grade applications [37,38]. By designing all-in-one materials incorporating these unique material properties, scientists have moved onto two known classes of polyesters with exceptional characteristics, such as thiophene-based biopolymers derived from 2,5-thiophene dicarboxylic acid (TDCA), and furan-based biopolymers derived from 2,5-furandicarboxylic acid (FDCA), both of which have the potential to be 100% renewable. These materials have similar structures and properties to the petrochemical terephthalic acid (TPA), including excellent gas barrier properties, chain conformation, and molecular dynamics [31,39]. Although bio-based routes for the synthesis of TPA have been constantly refined over time, PET is still predominantly produced from fossil sources. In this study, special emphasis is placed on 1,6-hexanediol (1,6-HDO) as a primary diol derived from hexane substituted with hydroxy groups at positions 1 and 6 [40]. Of particular interest is 1,6-HDO, not only because of its recent bio-based origin, but also because it actively participates in the polymerization behavior of polyesters due to the considerable length of its methylene chain. In an industrial setting, 1,6-HDO is synthesized from 5-hydroxymethylfurfural (HMF) in a fixed bed reactor using double layer catalysts of Pd/SiO_2_ + Ir-ReOx/SiO_2_ under precise conditions (373 K, 7.0 MPa H_2_, in solvent mixtures of 40% water and 60% tetrahydrofuran (THF)), resulting in a 60% product with a significantly low carbon footprint [41]. Furthermore, 2,5-thiophenedicarboxylic acid (TDCA) is a bio-based aromatic diacid that is produced industrially from adipic acid (AA) and thionyl chloride via a multi-step process including bioconversion, dehydration, and oxidation, producing important intermediates such as succinic acid (SA). Recently, Guidotti et al. [42], Terzopoulou et al. [43], and Wang et al. [44] investigated a series of thiophene-based polyesters incorporating 1,6-HDO with enhanced biodegradability under the influence of varying enzyme activities such as cutinase, lipase, and esterase, and established a correlation between the degradability rate and 1,6-HDO amounts in the polyester structure. They found that the higher the 1,6-HDO moieties, the faster the degradation rate in soil under passive or enzymatic degradation. Djouonkep and coworkers [30,45], as well as Wang et al. [44,46], systematically synthesized a series of bio-based polyesters from TDCA, each incorporating 3–8 methylene chain-length diols. They noticed that the polyesters’ chain mobility and morphology strongly depended on 1,6-HDO mole ratios added at the polymerization stage to tune the material properties. Unfortunately, the above polymeric materials possessed some shortcomings, such as low processability and poor mechanical properties, which hindered their widespread applications. Similarly, Papadopoulos and coworkers [47] investigated a novel poly(hexylene carboxylates) (PHF) blend that seemed to crystallize faster and in more significant amounts than poly(propylene/butylene carboxylates) (PPF/PBF) during cooling below the melting point, but had a lower crystallinity degree and molecular weight due to a lack of aromatics in its structure.

This study presents the novel synthesis of poly(hexylene 2,5-thiophenedicarboxylate-co-bis(2-hydroxyethoxybenzene) (PTB_x_H_y_), by blending aromatic 1,4-Bis(2-hydroxyethyl)benzene (BHB) and aliphatic 1,6-hexanediol (1,6-HDO) diols with 2,5-thiophendicarboxylate (TDCA). The selection of TDCA and BHB was based on both their green origin, showing similar structural properties to TPA, as well as incorporating longer methylene carbon chains from 1,6-HDO. The copolyester properties were adjusted by varying the hexylene thiophenedicarboxylate (THH) content over 1,4-bis(2-hydroxyethyl)benzene thiophenedicarboxylate (TBB). The use of porcine pancreatic lipase (PP-L) demonstrated high affinity for the given copolyesters, with high degradation rates under specific conditions in the natural environment. The research is significant as it explores the structure–property relationships of copolyesters and provides a new method for sustainable material production.

## 2. Results and Discussion

### 2.1. Polymer Synthesis and Characterization

The molecular characteristics of thiophene-based copolyesters, PTHH, PTBB, and PTB_x_H_y_ were studied and the results are presented in Table 1. The semi-crystalline copolyesters were found to be light brown-colored solids that turned into white-colored floccules after purification. The addition of more diol units in the copolymerization reaction led to the full transesterification of the TDCA monomer and also resulted in termination with hydroxyl chains. Melt polymerization was used in the synthesis process as it allowed for faster composition changes in the molten state, leading to the formation of complex copolyesters in a 3-D form. It was also found that increasing the amount of 1,6-HDO units significantly reduced the copolymerization time, making the process more cost-effective. The reaction temperature was kept below 230°C to avoid yellowing side reactions such as etherification and thermal decomposition of TDCA-based polyesters. The pressure was gradually reduced to minimize oligomer sublimation and ensure the quality of the final product. To monitor the reaction process, ^1^H NMR was used, which showed that 1,6-HDO was consumed twice as fast as BHB in varying molar ratios. The final molar feed (Φ) of THH and TBB units in the copolyester matrices was estimated using Equation (1) and was found to be close to the values monitored by ^1^H NMR.

(1) $$\mathrm{Molar}\;\mathrm{Feed}\;\left(\Phi \right)=(I_{\mathrm{TDCA}\text{-}\mathrm{HDO}}{/}_{(I_{\mathrm{TDCA}\text{-}\mathrm{BHB}}}+I_{\mathrm{TDCA}})\times 100$$

In Equation (1), I_TDCA-BHB_ represents the integration (a, c) peaks of the TDCA-BHB segment; I_TDCA-HDO_ is the integration (a, d) peaks of the TDCA-HDO segment and I_TDCA_ is the integration of TDCA. The integration of the peaks (c and d) in the NMR spectra allowed the determination of the degree of randomness (R) of the copolyesters. The number average molecular weight (M_n_) ranged from 33.4 to 38.7 × 10^3^ g/mol, while the weight average molecular weight (M_w_) ranged from 67.4 to 78.7 × 10^3^ g/mol, with dispersity indices (Ð) ranging from 2.00 to 2.12, which is suitable for most applications, according to GPC/SEC, which separated the polymers based on their size, molecular weight, and shape. When the polymers are dissolved, they form a coil conformation that resembles a ball of string. Although they are chains, they appear as tiny spheres during GPC/SEC analysis, with the sphere size dependent on molecular weight. Higher molecular weight polymers form larger spheres and elute faster than their lower molecular weight counterparts. The M_w_ increased with increasing 1,6-HDO units, to a tipping point where further additions disrupted the integrity of the system, making it increasingly amorphous, resulting in a significant decrease in the viscosity as measured by the Ubbelohde viscometer.

### 2.2. FTIR and ^1^H NMR Analysis

**Figure 1:**
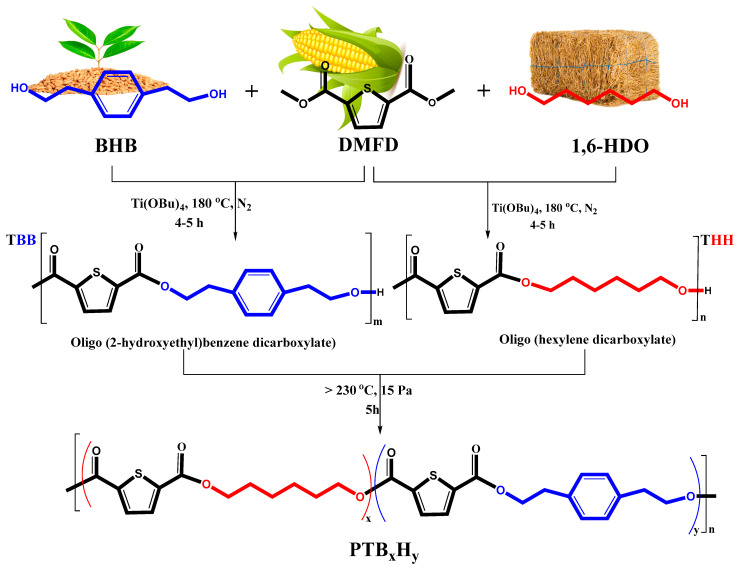
Synthetic route for poly(hexylene 2,5-thiophenedicarboxylate-co-bis(2-hydroxyethoxybenzene) (PTB_x_H_y_) copolyesters.

The FTIR spectra of the copolyesters PTBB, PTHH, and PTB_x_H_y_ were analyzed, and the results are presented in Figure 1. The spectra show the characteristic bands of the thiophene ring at 3120 cm^−1^, 1570–1580 cm^−1^, and 711 cm^−1^, which are attributed to C=C, C–H, and C–S stretch bonds, respectively [48,49,50,51]. The sharp peak of the phenyl ring was observed at 1577–1585 cm^−1^ and 750 cm^−1^, and the methylene groups attached to the THH and TBB units were seen at 2930 and 2865 cm^−1^, respectively. The strong absorption peaks at 1720 cm^−1^ are due to the C=O stretching vibration in the TDCA segment, and the peaks at 1270 and 1128 cm^−1^ are attributed to the symmetric and asymmetric O–C=O groups connecting the different segments of the polymers [52,53]. When the THH content was increased, the intensity of the bands corresponding to the CH_2_ groups increased, confirming the incorporation of the TBB and THH units into the structure of the copolymers. These results provide evidence of the successful synthesis of the copolymers, with the desired composition and molecular structure.

**Figure 2:**
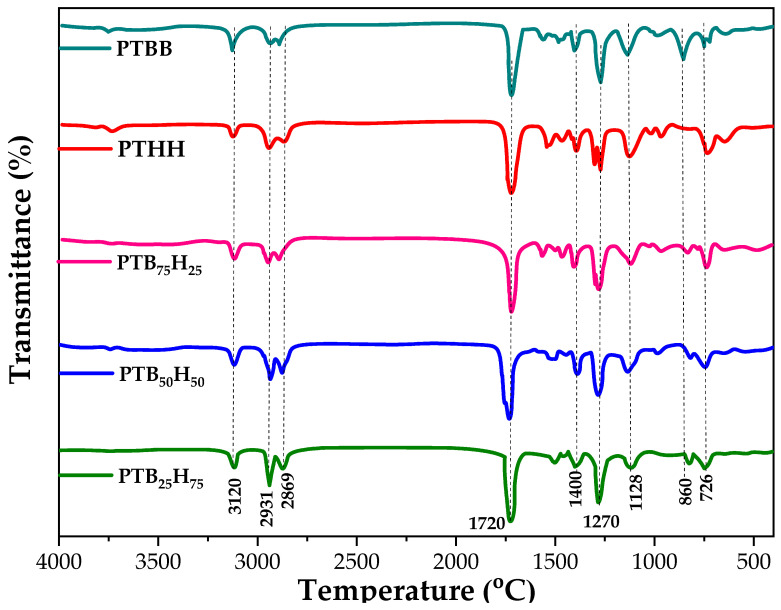
FTIR spectra of the homologs poly(1,4-bis(2-hydroxyethyl)benzene thiophenedicarboxylate) (PTBB), poly(hexylene thiophenedicarboxylate) (PTHH), and poly(hexylene 2,5-thiophenedicarboxylate-co-bis(2-hydroxyethoxybenzene) (PTB_x_H_y_) copolyesters.

The chemical structure and composition of the homologs and copolyesters were studied using ^1^H NMR spectra, which are presented in Figure 2. The spectra showed that the resonance peak corresponding to the thiophene ring peaks (a, 2H, s) appeared at 7.71 ppm with varying intensities, reflecting the differences in the kinetics of the diols. For the TBB unit, the peak of the phenyl ring (b, 4H, s) appeared at 7.11 ppm, and the thiophene-bound methylene protons (c, 4H, t) appeared at 4.63 ppm [54]. For PTHH, the methylene protons of the aliphatic subunit bounded to the thiophene moieties showed peaks between 4.22 and 4.41 ppm, while the methylene not bound to the thiophene moieties (e, f, 8H, m) appeared in the range of 1.21–2.01 ppm [55]. In PTB_x_H_y_, the assigned peaks (b, c and d) corresponded to the aromatic segment induced by TBB units, whereas the peaks (d, e and f) described the aliphatic characteristic of THH units shifting to lower values, typical of aliphatics (1,6-HDO). The composition of the copolyesters, as listed in Table 1, was found to be in good agreement with the peak representation, and no additional peaks were observed, suggesting that the homologs and samples were of good purity. These results provide further evidence of the successful synthesis of the copolymers with the desired chemical structure and composition.

### 2.3. Crystallinity Properties

**Figure 3:**
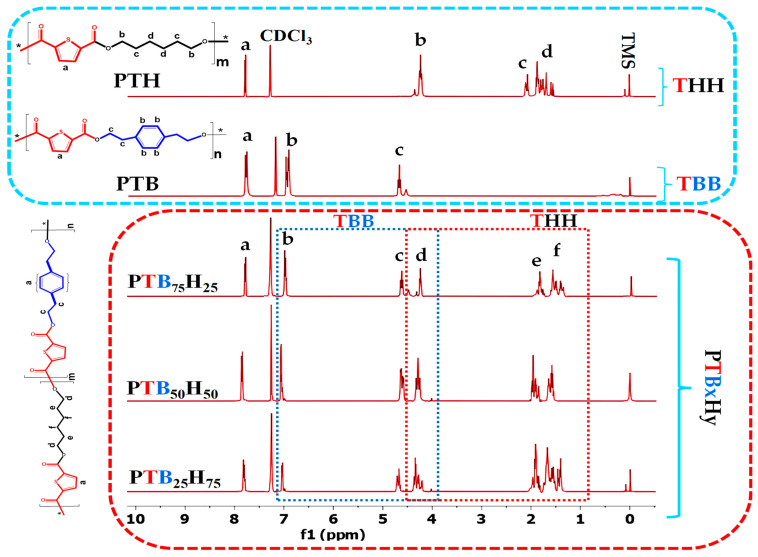
^1^H NMR spectra of poly(1,4-bis(2-hydroxyethyl)benzene thiophenedicarboxylate) (PTBB), poly(hexylene thiophenedicarboxylate) (PTHH), and poly(hexylene 2,5-thiophenedicarboxylate-co-bis(2-hydroxyethoxybenzene) (PTB_x_H_y_) copolyesters.

The semi-crystallinity of the thiophene–aromatic polyesters was corroborated by XRD analysis (Figure 3). The diffractograms followed a pattern similar to poly(hexamethylene carbonate)-glycol crystallography [56,57]. A complex pattern containing six characteristic peaks appeared at 2θ = 9.2°, 18.7°, 20.1°, 25.7°, 27.1°, and 28.2°. In the PTHH X-ray diffractogram, four sharp signals were displayed at 2θ = 9.1°, 18.7°, 25.9°, and 28.4°. PTBB, PTB_50_H_50_, PTB_75_H_25_, and PTB_25_H_75_ showed six signals with varying intensities, which indicated the different crystallinity degrees induced by their varying mole ratios. The sharp peaks suggested a high degree of macromolecular order and slowly decreased due to the addition of aliphatic methylene carbons. All the polyesters were clearly semi-crystalline, except for PTHH, which was more amorphous in composition but managed to display some crystalline peaks at 9.4°, 18.5°, and 25.7°, possibly attributed to TDCA unit. The PTB_50_H_50_, PTB_75_H_25_, and PTB_25_H_75_ copolyesters exhibited trades to PTBB, but with a lesser intensity by a factor of ¾, upon continuous addition of the THH unit. The crystal phase grew and accommodated the THH units up to a maximum of 75 mol%, after which the amorphous phase became dominant. Because the copolyesters possessed the network properties of both homologs, they can be called isomorphic. The morphology of the copolyesters changes from crystalline to amorphous, then back to semi-crystalline, offering satisfactory flexibility properties in the process.

### 2.4. Thermal Analysis and Properties

**Figure 5:**
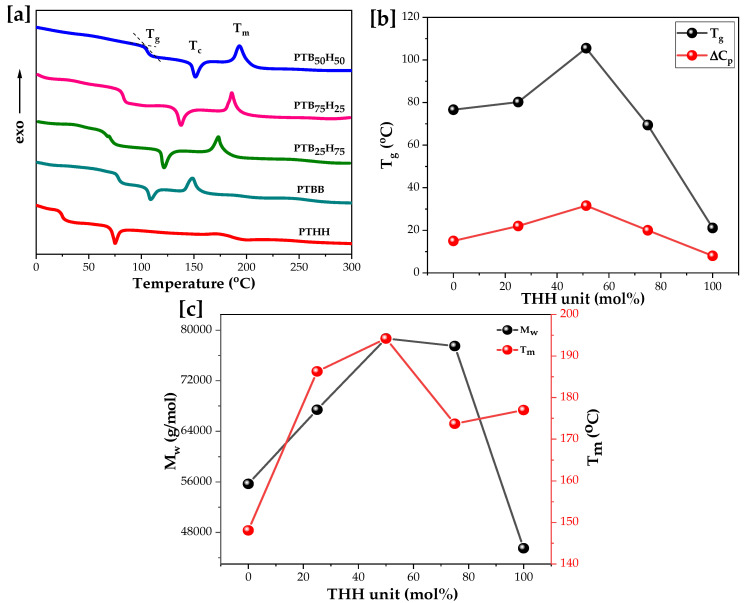
[**a**] DSC of poly(1,4-bis(2-hydroxyethyl)benzene thiophenedicarboxylate) (PTBB), poly(hexylene thiophenedicarboxylate) (PTHH), and poly(hexylene 2,5-thiophenedicarboxylate-co-bis(2-hydroxyethoxybenzene) (PTBxHy) copolyesters; [**b**] T_g_ and ΔCp vs. mol% of THH unit; and [**c**] M_w_ and T_m_ vs. mol% of hexylene thiophenedicarboxylate (THH) unit.

The thermal properties of PTBB, PTHH, and PTB_x_H_y_ materials obtained during the second heating and cooling runs are displayed in Table 2. The endothermic baseline deviation indicates the visible glass-to-rubber transitions, followed by an endothermic phenomenon associated with the melting of the crystalline phase, which is typical of semi-crystalline materials. The DSC analyses followed a heat/cool/heat procedure, where the first heating run was considered to erase the sample history. As shown in Figure 5a and Table 2, the T_g_ varied between 22.1 and 105.5 °C, depending on the chain stiffness, and increased with increasing THH units below 60 mol%. However, beyond 50 mol%, further addition of THH unit acts as a defect rather than a reinforcement to the copolyester matrixes, leading to decreasing crystallization enthalpy and molecular weight. Sharp crystallization and melting peaks were observed for all the samples except PTHH, which showed a slightly dominant amorphous phase due to the higher methylene number content of the aliphatic diols. The T_g_ value of PTB_50_H_50_ was slightly higher than that of PTB_75_H_25_ and PTB_25_H_75_, attributed to its high molecular weight, as seen in Figure 5b. For all PTB_x_H_y_, single melting and cold crystallization peaks were observed. The co-crystallization existed as a single phase in the copolyesters, where the crystallization rate decreased with increasing THH units. Compared with their homologs (PTBB and PTHH), PTB_x_H_y_ showed wider melting and melt crystallization peaks, possibly due to the concurrent crystallization of two different crystal patterns. The T_m_ of PTHH was approximately 28.9 °C higher than that of PTBB, while the T_g_ was 55.5 °C lower than that of PTBB, which proved the faster recrystallization from a melt of 1,6-HDO. Figure 5c shows the correlation between the T_m_ and M_w_ of the copolyesters. The addition of the THH unit initially strengthens the polyester chain segment via entanglement and increases the molecular weight, but as the 50 mol% range is exceeded the matrix blurs and the molecular weight decreases, because the ordered TBB-like cell units no longer hold the THH cell unit, gradually leading to a disordered pattern. Compared to FDCA-derived analogues such as poly(vanillin-co-ethylene furanoate), poly(ethylene 2,5-furandicarboxylate-co-ethylene terephthalate), and poly(decamethylene-co-isosorbide 2,5-furandicarboxylate) synthesized by Kasmi et al. [58], Knoop et al. [59], and Chebbi et al. [60], the T_g_ values, reported to be around 69.5, 38, and 20.6 °C, respectively, are much lower than the T_g_ of 105.5 °C measured for PTB_50_H_50_. Other existing copolyesters, such as poly(butylene 2,5-thiophenedicarboxylate) (PBTF) [46], poly(1,4-butylene-co-1,4 cyclohexane 2,5-furandicarboxylate) (PBCF) [39] and poly(propylene cyclohexanedicarboxylate) (PPCE) [61], demonstrated similar eutectic (V-shaped) behaviors.

In Figure 5a,b the TGA and dTG curves of the copolyester materials are presented. The results suggest that the thermal stability of copolyester materials increases with the increase in the THH units. This can be seen in the progression of degradation temperatures (T_d,5%_, T_d,50%_, and T_d,max_) from 325 °C to 366 °C, 365 °C to 400 °C, and 388 °C to 432 °C, respectively. The TGA curves followed a one-step thermal decomposition process with dTG under N_2_ atmosphere. The high thermal stability of thiophene-based copolyesters is attributed to the presence of rigid phenyl (BHB) and thiophene (TDCA) units in the polymer chains. [30], while the presence of 1,6-HDO enhanced the stretchability and flexibility of the copolyesters to some extent, in agreement with the findings of Wang et al. [49]. PTB_50_H_50_ and PTB_75_H_25_ have similar thermal properties to PET, due to the good rigidity and symmetry of the TDCA and BHB rings and the pliability of the aliphatic methylene, with T_g_, T_d,5%_, and T_d,max_ of 79.0 °C, 407.3 °C, and 440.0 °C, respectively. For FDCA-based polyesters like poly(ethylene furanoate) PEF and poly(butylene furanoate) PBF, T_d,5%_ and T_d,max_ are found in the ranges of 315 to 367 °C and 378 to 405 °C, respectively [62,63]. This is slightly lower than our synthesized copolyesters, because the polarization and non-linear axis of rotation in furans prevent the ring from flipping. Similarly, the longer C–S–C bond in thiophenes compared to C–O–C contributed to this phenomenon [31,64].

### 2.5. Gas Barrier Properties

**Figure 7:**
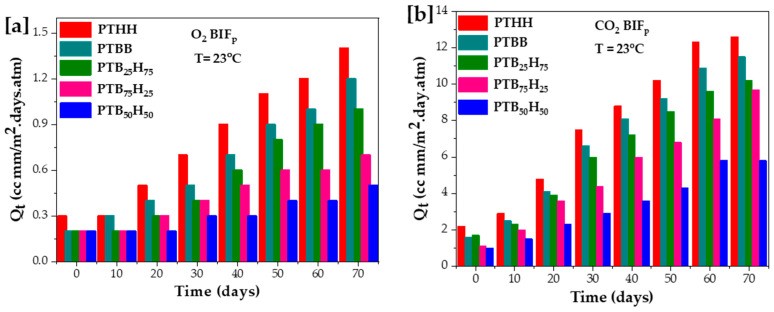
[**a]** O_2_ BIFp and [**b**] CO_2_ BIFp for the poly(1,4-bis(2-hydroxyethyl)benzene thiophenedicarboxylate) (PTBB), poly(hexylene thiophenedicarboxylate) (PTHH), and poly(hexylene 2,5-thiophenedicarboxylate-co-bis(2-hydroxyethoxybenzene) copolyester films.

The semi-crystalline films were melt-pressed into thin films and tested on a gas permeability tester (Labthink VAC-V2, Medford, Massachusetts, USA UK). The morphology and degree of entanglement of the multiphase (semi-crystalline) affected the diffusion path of the permeating molecules [19,48]. The permeability coefficients were determined according to the ASTM D3985 standard and measured at 23 °C. The O_2_ barrier improvement factor (BIFp) was calculated by dividing the O_2_ permeability coefficient of the target polymer by the O_2_ permeability coefficient of PET over a period of 70 days. From previous studies [45,65], the thiophene ring, owing to its asymmetric rigid structure and lower dipole moment of 0.51D, significantly impacted the gas barrier properties of polyesters compared to furan (dipole moment of 0.70D). Herein, PTB_50_H_50_ displayed the lowest permeability to O_2_ and CO_2_, with a coefficient value of 0.47 and 3.02, respectively, attributed to its high molecular weight and T_g_ value. As the THH unit increases, the BIF_O2_ and BIF_CO2_ significantly increase, reaching an average value of 1.41 and 12.32 for PTHH, respectively. Because of the dominant crystalline structure of TBB units compared to THH units, the PTB_x_H_y_ copolyesters demonstrated better O_2_ barrier properties than poly(butylene-adipate-*co*-terephthalate) (PBAT) [66], poly(lactic acid) (PLA) [67], and PET [37], organising the properties in the order of PTB_50_H_50_ > PTB_75_H_25_ > PTB_25_H_75_. However, as the THH units exceeded 60 mol%, the crystalline structure of the polymer became more randomized, allowing for larger interspaces in the polymer matrix, which resulted in higher gas permeation, as seen in Figure 7.

### 2.6. Dynamic and Mechanic Properties

**Figure 8:**
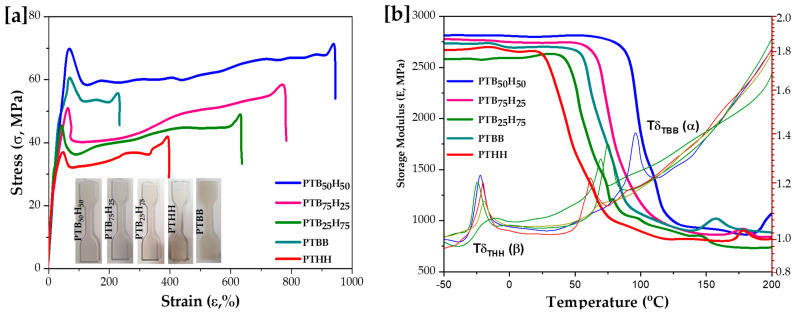
[**a**] Stress–strain curves and [**b**] DMTA for thepoly(1,4-bis(2-hydroxyethyl)benzene thiophenedicarboxylate) (PTBB), poly(hexylene thiophenedicarboxylate) (PTHH), and poly(hexylene 2,5-thiophenedicarboxylate-co-bis(2-hydroxyethoxybenzene) copolyester materials.

The mechanical properties, obtained by dynamic mechanical thermal analysis (DMTA), and stress–strain curves were obtained after the samples were compression molded from poly(ether-ester), as shown in Figure 7. The tensile stress (σ), tensile strength (Ɛ), Young’s modulus (E), and Tan δ data are summarized in Table 3. As shown in Figure 8a, the tensile properties of PTB_50_H_50_ were 70.5 MPa for strength and 950% for the elongation at break, which are close to those of PET, at 84.5 MPa and 1500% [45]. PTB_75_H_25_ showed better mechanical performances than PTB_25_H_75_ due to its higher TBB content. PTBB displayed higher stress (62.5 MPa), compared to PTB_25_H_75_ (46.4 MPa) and PTB_75_H_25_ (52.9 MPa), due to its higher TBB units. However, PTBB exhibited lower strain (237%) compared to those of PTB_25_H_75_ (641%) and PTB_75_H_25_ (785%), due to the higher THH content. The inflexibility of the thiophene-based copolyesters followed the order of PTBB > PTHH > PTB_25_H_75_ > PTB_75_H_25_ > PTB_50_H_50_.

The main transitions observed were α- and β-transitions, when approaching the glass transition temperatures of the copolyesters. Compared to the T_g_ values determined by DSC analysis (Table 2), the DMTA curves show a rough correspondence with transition temperatures at the initial loss modulus of the copolyesters. Two distinct regions were identified [68], a glassy high modulus region where the segmental mobility is restricted, and a transition zone where a significant loss in modulus is observed due to elasticity cleavage. Knowing that the T_g_ of semi-crystalline polyesters is sensitive to their crystallinity, the α-transition was observed at around −15.55 °C and attributed to the THH motif, while the β-transition was observed between 50–100 °C and corresponded to the chair-chain flip transitions of TBB units in polymer chains. These transitions are generally correlated with the appearance of movements of side chains or of functional groups in the polymers, originating from the co-crystallization of the TBB and THH units. However, further research is necessary to fully understand the frequency of these transitions by comparing the block polymerization to the random polymerization of the copolyesters.

### 2.7. Degradability Studies

**Figure 9:**
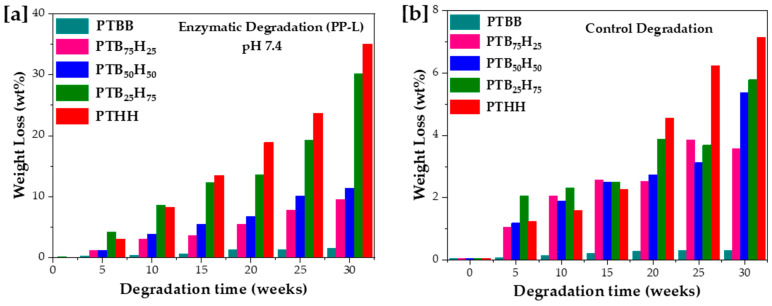
[**a**] Enzymatic degradation and [**b**] passive hydrolysis for the poly(1,4-bis(2-hydroxyethyl)benzene thiophenedicarboxylate) (PTBB), poly(hexylene thiophenedicarboxylate) (PTHH), and poly(hexylene 2,5-thiophenedicarboxylate-co-bis(2-hydroxyethoxybenzene) copolyesters.

A critical factor in the development of green polymeric polyesters is their ability to biodegrade after their end of life in the natural environment [30,46,69]. Due to its environmental friendliness and low processing cost, enzymatic degradation has gradually gained popularity as the method of choice for plastic waste recycling. Within this group of thiophene-based polyesters, the hydrolyzable ester bonds and the C–S bonds of thiophenes play an essential role in the ignition process [31]. This enzymatic degradation follows a two-step process in which extracellular enzymes produce microorganisms (mostly sulfur-loving bacteria) that induce hydrolysis of ester bonds, resulting in the cleavage of the polymer chains into water-soluble hydrolysates such as monomers and oligomers. Afterwards, the microorganisms metabolize the water-soluble hydrolysates, in a process known as mineralization, into by-products such as methane, CO_2_, and H_2_O [19,30,70]. The enzymatic biodegradation is strongly correlated to properties such as chain composition, molecular weight, and crystallinity of the copolyesters [47,71,72]. Herein, the in vitro degradation was evaluated over a period of 30 weeks, with and without the use of porcine pancreatic lipase, at 37 °C in phosphate buffer at pH 7.4. As shown in Figure 8a, PTHH could reach a degradation of 35 wt% after 30 weeks, possibly due to the larger methylene segments that are easily hydrolyzed by enzymes. PTBB displayed very little degradation, which was attributed to the steric hindrances of the bulky phenyl groups to hydrolysis. For the copolyesters, the increase in THH units considerably increased the degradation rate to values such as 9.6, 11.4, and 30.2 wt% for PTB_75_H_25_, PTB_50_H_50_, and PTB_25_H_75_, respectively. The results of the normalized control experiment without enzymes are displayed in the Figure 9b. The weight loss was limited due to the chemical inertness displayed by the copolyesters observed at 3.6, 5.4, and 5.8 wt% attributed to PTB_75_H_25_, PTB_50_H_50_, and PTB_25_H_75_, respectively. Interestingly, the degradation rate of PTHH is higher than that of the copolyesters, due to its lower crystallinity in all media.

From the SEM analysis presented in Figure 9, PTBB clearly showed its limited degradation capacity; instead, the surface had visible cracks attributed to its high aromatic-induced stiffness. On the other hand, the other copolyesters showed a progressive degradation via microcavities and erosion on their surfaces, which is the result of abiotic and biotic hydrolysis over time. The enzymatic degradation cleaved the polymeric bonds connecting the monomeric units via microbial-to-substrate activity, leading to the progressive deterioration of the polymeric chain network. The SEM imaging revealed that the copolyester surfaces exhibit micropores and cavities, which deepen over time as degradation progresses. These findings suggest that porcine pancreatic lipase PP-L has good affinity for the polyesters and effectively breaks the polymeric bonds that connect the monomeric units. The presence of progressive deterioration on the surfaces is a visual representation micro-enzyme activity to macro-activity in the polymeric network and further confirms the efficacy of PP-L as a degradation agent. By using enzymatic degradation, the bio-based monomers in the polyester can be re-used to make new polymers, reducing the amount that is discarded into the environment. This environmentally sustainable process is a key aspect of the circular economy, where waste is reduced, and resources are conserved through closed-loop production systems.

### 2.8. Relevance of the Current Research for Practical Implementation

A practical application of this research lies in the development of biodegradable, high-performance plastic products using thiophene-based waste monomers for the synthesis of bio-based copolyesters. For example, the high tensile strength and toughness of these copolyesters could make them suitable for use in a wide range of products, from food packaging to agricultural mulch film. Additionally, the controlled crystallinity of these copolyesters, as well as their similar structure-to-properties with petrochemical-based polymers, could make them a suitable alternative to petroleum-based plastics. This could reduce dependency on finite fossil resources and reduce the impact of plastic waste on the environment. Moreover, the biodegradability of these copolyesters, when degraded with porcine pancreatic lipase (PP-L), could provide a sustainable solution for plastic waste management. The degradation of these copolyesters could lead to the recovery of monomers that can then be recycled and re-used to make new plastic products, instead of being discarded into the environment. Thus, this research has the potential to provide practical solutions for the development of environmentally friendly and sustainable plastic products, as well as a more sustainable plastic waste management approach.

## 3. Materials and Methods

### 3.1. Materials

We purchased 2,5-thiophene dicarboxylic acid (TDCA) (purity > 99.5%), 1,6-hexanediol (1,6-HDO, 99%), and 1,4-Bis(2-hydroxyethyl)benzene (BHB, 99%) from Aladdin Reagent Co. Ltd., Shanghai, China. Titanium butoxide (Ti(OBu)_4_) (purity > 97.0%) was purchased from Sigma Aldrich Chemical Co., Ltd. (Shanghai, China). Solvents such as methanol, ethanol, and chloroform (purity > 99%) were purchased from Sinopharm Chemical Reagent Co. Ltd., Zhejiang, China. Porcine pancreatic lipase (PP-L) with activity > 55 U/mL was purchased from Sigma-Aldrich, Taufkirchen, Bavaria State, Germany, and cultured in a dark, humid container for a period of time prior to its utilization.

### 3.2. Methods

#### 3.2.1. Characterization Techniques

The physico–chemical and mechanical properties of the synthesized copolymers were studied using the characterization techniques described below.

##### Fourier-Transform Infrared Spectroscopy (FTIR)

The chemical structure of the copolymers and homologs was confirmed by FTIR analysis on a Bruker VERTEX 70 infrared spectrometer (Ettlingen, Germany). The spectra were recorded over a range of 4000–400 cm^−1^ with 32 scans and a resolution of 4 cm^−1^.

##### ^1^H NMR Nuclear Magnetic Resonance

A Bruker Avance DMX NMR instrument (Leipzig, Germany) at room temperature at 600 MHz with tetramethylsilane (TMS) as the standard reference was used to perform ^1^H NMR spectroscopy. Each sample was previously dissolved in deuterated chloroform with a 10 mg/mL concentration.

##### Gel Permeation Chromatography (GPS)/Size Exclusion Chromatography (SEC)

The number-average molecular weight (M_n_), weight-average molecular weight (M_w_), and dispersity (Đ) were obtained by gel permeation chromatography/size exclusion chromatography (GPC/SEC) performed on a Viscotek TDAmax G gel chromatography analyzer (Worcestershire, U.K.). The GPC/SEC system operates by pumping DMF solvent (mobile phase) through the column, evaluated against standard distributed polystyrene at a flow rate of 1.0 mL/min at 40 °C. The solvent carries the sample and separated components through the column and to the detector for analysis.

##### Thermal Analysis

Differential scanning calorimetry (DSC) and thermogravimetric analysis (TGA) were performed under inert atmosphere (N_2_ flux of 30 mL/min) with a Netzsch STA449 F3 Jupiter DSC instrument and a PerkinElmer TGA7 (Leipzig, Germany), respectively. For TGA, about 5 mg of polymers were heated at a heating rate of 10 °C/min with a temperature range of 25 °C to 700 °C, while for DSC, about 8 mg were heated at a rate heating at 20 °C/min from room temperature to 200 °C above the melting point.

##### X-Ray Diffraction (XRD)

X-ray diffraction (XRD) patterns were obtained using a Rigaku Co. (Tokyo, Japan) MiniFlex II XRD system with CuKα radiation (0.154 nm) in the 10–50° range of 2-theta at a scan rate of 0.02°/min.

##### Scanning Electron Microscopy (SEM)

The morphology of the freeze-dried polyester samples was studied with a JEOL (Tokyo, Japan) JSM 7610F field emission scanning electron microscope (SEM) at an accelerating voltage of 10 kV.

##### Gas Barrier Properties

The oxygen transmission rate (OTR), permeability, and barrier improvement factor were measured on a Labthink VAC-V2 gas permeability tester (Germany) over a downstream coulometric sensor of ASTM D3985 at 23 °C. The films, with a surface area of approximately 38.5 cm^2^, were melt-pressed and placed between two chamber plates. One chamber was filled with O_2_ gas (P = 0.1001 MPa, T = 23 °C; gas stream = 100 cm^3^/min; with relative humidity varying from 0 to 85%), while the other was filled with CO_2_ gas. Triplicate measurements were taken for the entire test and the mean values as well as standard deviations were reported.

##### Microtensile Testing

The stress–strain properties of the copolyesters were measured on an Instron 5544 tester (T.A., Hudson, MA, USA) after the samples were compressed and molded into dumbbell-shaped strips with a model 3889 heat press (Carver Inc., Ontario, NY, USA) with a 500 N load cell. Cast specimens were tested according to ASTM D412a rules. For the standard deviations, two sets of values were recorded.

The biodegradation of the synthesized copolymers was studied using both passive and enzymatic pathways. The experimental conditions used in each of the biodegradation processes involved are described below.

#### 3.2.2. Passive and Enzymatic Degradations of Copolyesters

Passive (control) and enzymatic solutions were employed to evaluate the systematic degradability of the copolyesters under varying conditions. Films of specific shapes (0.3 mm thickness) were incubated in phosphate-buffered solution (pH 7.4) at 37 °C, with and without (for the control experiment) 0.2 mg/mL of porcine pancreatic lipase (PP-L) for 30 weeks in a dark environment. The pH was monitored using a pH meter (Hong Kong, China), and the enzyme solution was carefully replaced every 3 days to maintain total enzymatic activity. The vials were removed every 7 days, gently rinsed with distilled water and methanol (to inhibit enzyme activity), and dried under vacuum until they reached a constant weight. The extent of degradation (weight loss) was estimated before and after the hydrolysis reaction according to Equation (2).

(2) $$\mathrm{Weight}\;\mathrm{loss}\;\left(\%\right)=\frac{W_{0}-{\;W}_{i}}{W_{0}}\times 100$$

where W_o_ and W_i_ are, respectively, the initial mass of the sample (in mg) and the mass of the sample at time under study.

Intrinsic viscosities ([η]) were determined using an Ubbelohde viscometer at 30 °C. The mixture of tetrachloroethane and phenol (1:1, w/w) was used as solvent and dissolved 125 mg of PTB_x_H_y_ samples. The average rate of outflow for the solvent was measured as t_0_, and the average rate of outflow for the sample was measured as t_1_, which was found using Equations (3) and (4):

(3) $$\eta_{\mathrm{sp}}=\frac{t_{1}-t_{0}}{t_{0}}$$

(4) $$\left[\eta \right]=\frac{\sqrt{1+1.4\eta_{\mathrm{sp}}}-1}{0.7C}$$

where C is the concentration of the solution, and t_0_ and t_1_ were the flow time of solvent and solution, respectively.

### 3.3. Preparation of Poly(hexylene thiophenedicarboxylate) (PTHH), Poly(1,4-bis(2-hydroxyethyl)benzene thiophenedicarboxylate) PTBB and Poly(hexylene 2,5-thiophenedicarboxylate-co-bis(2-hydroxyethoxybenzene) PTB_x_H_y_ Copolyesters

**Figure 10:**
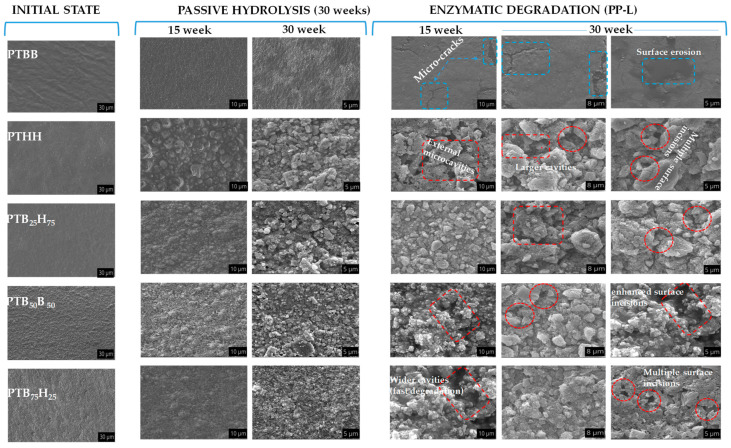
SEM micrographs of thepoly(1,4-bis(2-hydroxyethyl)benzene thiophenedicarboxylate) (PTBB), poly(hexylene thiophenedicarboxylate) (PTHH), and poly(hexylene 2,5-thiophenedicarboxylate-co-bis(2-hydroxyethoxybenzene) copolyester freeze-dried samples.

Prior to the synthesis of the copolyesters, esterification of 2,5-thiophenedicarboxylic acid under excess methanol and drops of sulfuric acid (H_2_SO_4_) heated to 70 °C gave dimethyl 2,5-thiophenedicarboxylate according to the ready-to-use method described in the literature [45]. The preparation of the copolyesters followed a two-step melt polymerization as an easy-to-green semi-continuous process to yield the random biobased copolyesters. The synthetic setup consisted of a 250 mL steel reactor attached to a water condenser, stirrer, vacuum pump, and N_2_ gas inlet. TDCA (carboxylate) was added to the reactor along with varying amounts of aromatic and aliphatic diols in a molar ratio of 3:2:2, with Ti(OBu)_4_ (0.5% relative to TDCA) as catalyst. Before the reaction, the reactor was purged several times with N_2_ to eliminate air. The optimal molar ratio was initially determined via an orthogonal test, where the sum of the diols slightly above the diacid is ideal for the polyester chains to be hydroxyl groups terminated. The esterification step was fully achieved below 160 °C at constant N_2_ flow for 5 h. The temperature was then raised to at least 180 °C, but not higher than 240 °C, for 4–6 h to produce the respective copolyesters via the polymerization stage. This process was operated above the transient air pressure to enhance the release of water and methanol droplets. The droplets were condensed and distilled out periodically to prevent oxygen from re-entering the reactor. Monitoring the process via the stirring torque was an excellent way to determine the end of the reaction. The homologs hexylene thiophenedicarboxylate (THH) and 1,4-bis(2-hydroxyethyl)benzene thiophenedicarboxylate (TBB) were synthesized from the same process. Finally, the polymers were purged out of the reactor and cooled to ambient temperature. They were later dissolved in excess chloroform and precipitated in cold methanol/ethanol (1:1 *v*/*v*) until the desired polymer product was purified, then freeze-dried at −70 °C for 12 h. The yield of purified polymers varied from 85 to 92%. After drying, the samples used for some physico-chemical characterizations were ground into powder, while the samples used for the tensile tests were cut into dumbbell-shaped bars. The synthesis route of the copolyesters is shown in Figure 10.

## 4. Conclusions

The results of this study demonstrate the viability of producing environmentally friendly thiophene-based copolyesters through the synergistic effects of 1,6-HDO and phenyl ring-based monomer (BHB). The copolyesters obtained were of high molecular weight, chemically inert, and had good thermal and mechanical properties, suitable for packaging applications. The ability to adjust the morphological characteristics, crystallinity and packing of the matrix can be quantified by the insertion of a THH unit, thereby reducing the cost of synthesis. The copolyesters exhibited high glass transition temperatures of 69.4 to 105.5 °C and, depending on the chain segment, melting points ranged from 173.7 to 194.2 °C. The mechanical properties of tensile strength, elongation at break, and storage modulus ranged from 46.4 to 70.5 MPa, 641 to 950%, and 1626 to 1665 MPa, respectively. The O_2_ and CO_2_ of the copolyesters were satisfactory for most uses, with PTB_50_H_50_ showing values around 0.47 and 3.02 Qt, which is better than PET and most furan-based polyesters. These qualities are crucial for uses requiring self-degradation of end-of-life materials. The thiophene-based polyesters were very susceptible to enzymatic degradation (>35% weight loss), except for PTBB at pH 7.4. The research highlights the importance of continued efforts to create renewable, efficient, and cost-effective methods to support and replace conventional monomers to achieve a sustainable economy. One method of challenging and reducing microplastics waste around the world requires scientists to conduct cutting-edge research on how to grow new microorganisms capable of breaking down plastics.

## Figures and Tables

**Figure 4:**
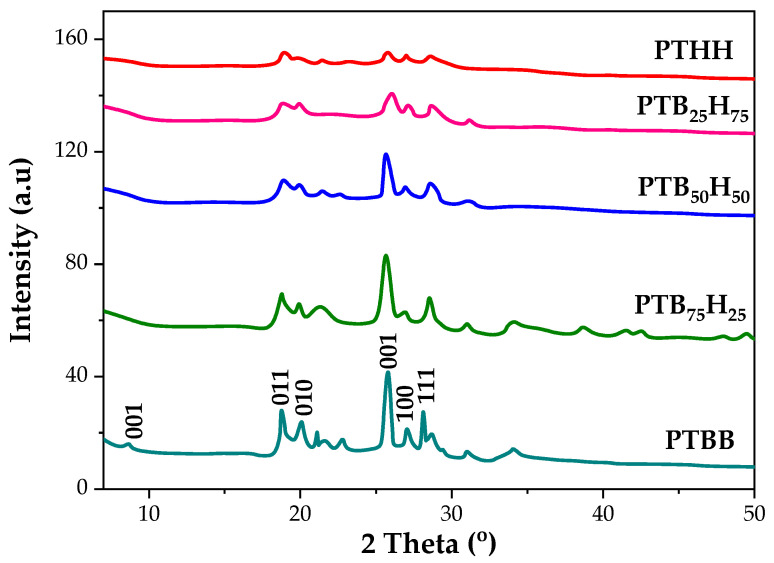
X-ray diffractogramms of poly(1,4-bis(2-hydroxyethyl)benzene thiophenedicarboxylate) (PTBB), poly(hexylene thiophenedicarboxylate) (PTHH), and poly(hexylene 2,5-thiophenedicarboxylate-co-bis(2-hydroxyethoxybenzene) (PTB_x_H_y_) copolyesters.

**Figure 6:**
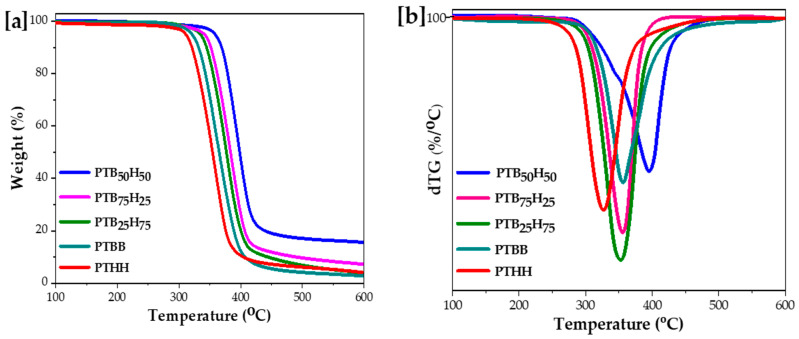
([**a**] TGA and [**b**] dTG of poly(1,4-bis(2-hydroxyethyl)benzene thiophenedicarboxylate) (PTBB), poly(hexylene thiophenedicarboxylate) (PTHH), and poly(hexylene 2,5-thiophenedicarboxylate-co-bis(2-hydroxyethoxybenzene) copolyesters.

**Table 1:** Feeding ratio, reaction condition, molecular weight (M_w_ and M_n_) and polydispersity index (Ð), intrinsic viscosity [η], and degree of randomness (R) of the copolyesters.

Samples	TDCA	BHB	HDO	Esterification		Polycondensation		Molar Feed (Φ) ^a^ BHB:HDO	GPC ^b^			[η] ^c^ (dL/g)	R
				Temp. (°C)	Time (h)	Temp. (°C)	Time (t)		M_n_ (g/mol)	M_w_ (g/mol)	Ð		
PTBB	1	1.5	-	160	4.0	230	3.0	98.7	25,500	55,700	2.18	1.04 ± 0.1	0.89
PTHH	1	-	1.5	160	3	210	4	97.3	21,300	45,500	2.14	0.95 ± 0.1	0.94
PTB_75_H_25_	1	1	0.5	180	5	220	4.0	64.5:35.5	33,400	67,400	2.02	1.15 ± 0.1	0.98
PTB_50_H_50_	1	0.75	0.75	180	4	220	4.0	45.3:54.7	37,200	78,700	2.12	1.21 ± 0.1	0.99
PTB_25_H_75_	1	0.5	1	180	4	210	3.5	33.8:66.2	38,700	77,500	2.00	1.10 ± 0.1	0.97

^a^ Calculated by the formula of Φ; ^b^ Determined by GPC in DMF solution with polystyrene standards; ^c^ Intrinsic viscosity obtained with an Ubbelohde viscometer at 25 °C.

**Table 2:** Thermal characterization of poly(hexylene 2,5-thiophenedicarboxylate-co-bis(2-hydroxyethoxybenzene) (PTB_x_H_y_) copolyesters and their homologs.

Samples	DSC				TGA			
	T_m_ [°C]	T_g_ [°C]	ΔC_p_ [J/g.K]	T_c_ [°C]	T_d,5%_ [°C]	T_d,50%_ [°C]	T_d,max_ [°C]	R_600_ [wt%]
PTHH	177.0	22.1	8.0	75.5	325	365	388	17.7
PTBB	148.1	76.6	15.3	138.3	334	374	402	15.4
PTB_75_H_25_	186.3	80.2	22.7	137.8	355	395	421	9.5
PTB_50_H_50_	194.2	105.5	25.5	138.3	366	400	432	4.9
PTB_25_H_75_	173.7	69.4	20.2	121.6	345	385	415	12.3

**Table 3:** Mechanical properties of PTB_x_H_y_ and its homologs.

Samples	E (MPa)	σmax (MPa)	Ɛmax (%)	Tan δ
PTB_50_H_50_	1665 ± 7	70.5 ± 1.1	950 ± 6	101.1 ± 0.06
PTB_75_H_25_	1644 ± 7	52.9 ± 1.2	785 ± 5	76.4 ± 0.09
PTB_25_H_75_	1626 ± 6	46.4 ± 1.4	641 ± 3	62.8 ± 0.04
PTBB	1594 ± 9	62.5 ± 1.1	237 ± 7	69.6 ± 0.05
PTHH	1545 ± 8	37.7 ± 1.3	398 ± 4	−14.6 ± 0.08

## Data Availability

Not applicable.

## Sample Availability

Samples of the compounds are available from the authors.
